# Supplementary material for: Bifidobacterial Transfer from Mother to Child as Examined by an Animal Model
Source: Microorganisms. 2019 Aug 27;7(9):293. doi: 10.3390/microorganisms7090293 (PMC6780879; doi:10.3390/microorganisms7090293)
Supplement: Supplementary file 1 [file microorganisms-07-00293-s001.pdf]

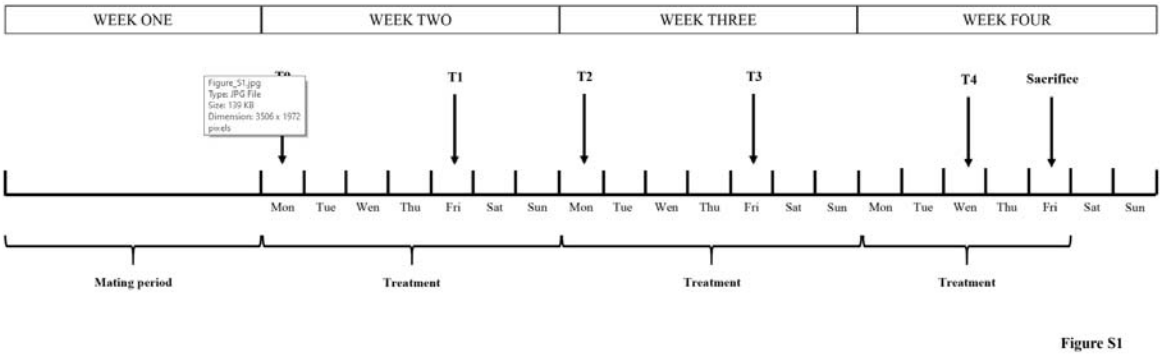

Figure 1. Timeline and general features of experimental procedure. This figure shows the schedule of the experimental procedures.

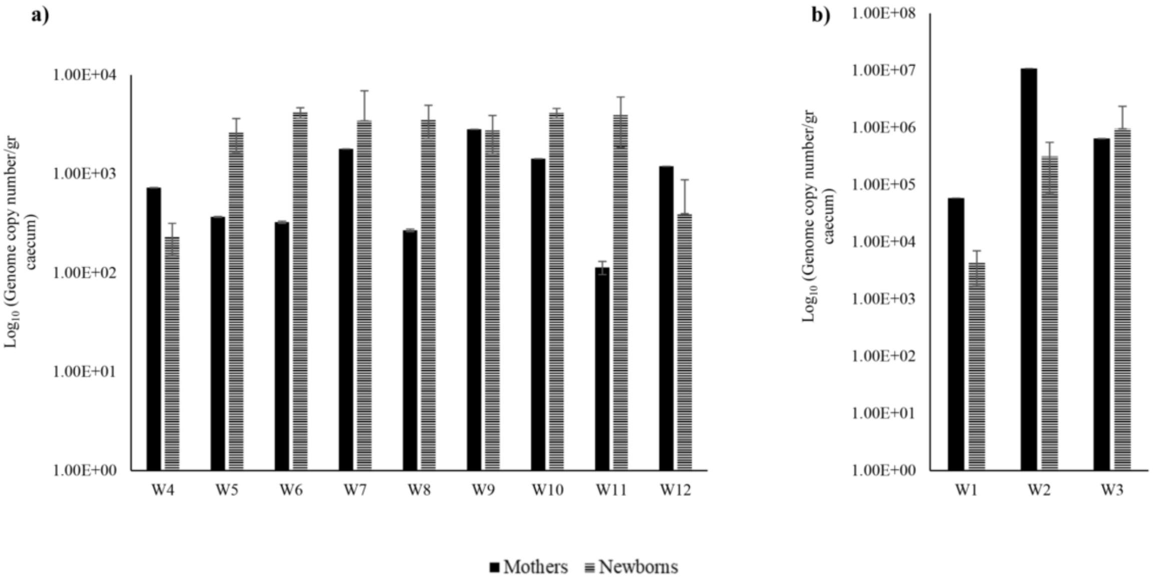

Figure 2. Schematic comparison of *B. bifidum* PRL2010 cell load in mothers caecum respect to newborns for each group. Panel a display the PRL2010 load of mothers PG vs. newborn PG. Panel b shows the PRL2010 load of mothers MCG vs. newborn MCG.
